# Physiological responses and histological alterations induced by pollution in the Nile tilapia from the Rosetta branch of the River Nile, Egypt

**DOI:** 10.1038/s41598-025-28119-x

**Published:** 2025-12-02

**Authors:** Sally M. Salaah, Ayat Taha, Fareda Medhat, Marwa M. El-Naggar

**Affiliations:** 1 National Institute of Oceanography and Fisheries, NIOF, Cairo, Egypt; 2 Department of Zoology, Faculty of Science, Ain Shams University, Abbassia, Cairo, 11566 Egypt

**Keywords:** Nile tilapia, Histopathology, Climate change, Cortisol, Thyroid hormones, Antioxidant activity, Ecology, Ecology, Environmental sciences, Zoology

## Abstract

The Nile River is the primary source of freshwater in Egypt and supports nearly all the anthropogenic activities in the region and, consequently, it is highly susceptible to pollution from diverse sources. Aquatic ecosystems in the Nile basin face increasing threats originated from pollution and seasonal fluctuations, both of which can profoundly affect the health and resilience of fish populations. Hence, addressing this issue is a must. This study investigated the impact of heavy metals (Fe, Zn, Cu, Cr and Cd) on key physiological and biochemical parameters for the Nile tilapia, *Oreochromis niloticus* sampled from two sites on the Rosetta Branch of the River Nile: El-Qanater (reference site) and El-Qatta (polluted site) during summer and winter of 2023. In addition, histological analysis was conducted on vital organs covering the winter season. Both site and season were detected with impacts on the hormonal concentrations in the fish samples. Compared to the reference site, fish individuals from the polluted site exhibited pronounced alterations in hormonal balance, with elevated cortisol levels and a marked decline in thyroid hormones (T3 and T4), while TSH levels were increased, particularly during winter. Elevated serum glucose, total protein, and albumin levels were evidenced for metabolic stress. Liver enzyme activity (ALT and AST) and kidney function biomarkers (urea, uric acid, and creatinine) were significantly increased, especially in winter. Profound responses to pollution were recorded with higher levels of SOD, CAT, GPx, and GST, aligned with a substantial decline in GSH levels. Moreover, higher malondialdehyde (MDA) levels were detected in winter. For fish organs histologically examined, a severe damage was remarkably observed in liver, gills, and kidney of fish from El-Qatta site. This study reveals the combined impact of pollution and seasonal changes on the Nile tilapia health, emphasizing the need for continuous monitoring and effective pollution control. Implementing targeted water quality programs in the Rosetta Branch is recommended to preserve fish health, biodiversity, and sustainable fisheries.

## Introduction

 In Egypt, the Nile River is the country’s primary resource of freshwater. As it flows northward beyond Cairo, it bifurcates at El-Qanater El-Khayria into two main branches (Damietta and Rosetta branches). However, the Nile River and its streams face severe ecological challenges due to pollution^1^, originated from effluents received from multiple point and non-point sources causing denaturation of the river water quality^2^. The increasing pollution from heavy metals (HMs) induces a substantial threat to aquatic ecosystems worldwide. These pollutants enter water bodies from numerous anthropogenic sources, including sewage discharge, industrial waste, recreational activities, and hospital effluents^3–5^. HMs are the most frequent pollutants in the River Nile, having the potential to disrupt the balance of aquatic ecosystems, adversely impacting fish health and biodiversity^6,7^. While certain HMs, such as Fe, Cu, and Zn, serve as crucial micronutrients, while their excessive accumulation can be toxic. On the other hand, metals like Pb, Hg, Cd, As, and Cr are highly toxic and carcinogenic. Even at low concentrations, Pb, Cd, and Cr exhibit significant toxicity^8,9^.

HMs are durable, accumulating in aquatic environments and entering the food chain, where they pose risks to aquatic life and human health^7,10^. Chronic exposure to HMs adversely impacts fish health, their physiological and metabolism processes, as well as fish reproductive functions. The severity of HM toxicity depends on the metal type, concentration, and duration of exposure^11,12^. In fish, the detoxification processes of HMs demand an intensive energy, shifting energy from being consumed in growth and development^13^. HMs penetrate fish body through multiple routes viz., the gills, digestive system, and skin, and eventually accumulate in edible tissues. This, in turn, limits fish nutritional benefits and may even pose pronounced risks to human health being the end consumer^10^. In fish, hormones play a part in numerous vital processes such as metabolism, osmoregulation, somatic growth, maturation, reproduction, and behaviour^14^. Moreover, it was reported that HM bioaccumulation in the Nile tilapia is associated with several endocrine dysfunctions^15^. It was deduced that a single and combined metals exposure remarkably declined the hormone levels, enhancing sex hormones receptor with a substantial effect on the level of ovarian metabolites. Furthermore, compared to the single exposure, HMs co-exposure led to extensive damaging ramifications.

The Nile tilapia is a widely farmed species, especially in Egypt due to its adaptability, tolerance, and ease of breeding. Eminently, the status of the physiological and antioxidant systems in this fish serves as reliable bioindicators for assessing water quality and identifying pollution levels^16^. Blood biochemical indices in fish are considered a significant dial of overall health status. These parameters mirror the vital organs’ efficiency including the gills, liver, and kidney. Variations in biochemical profiles have frequently been detected as a response to ecological stressors such as pollution, temperature changes, or hypoxia^17^. Consequently, monitoring the blood biochemical indices provides an acute insight into the physiological condition of fish^16^. Remarkably, fish histological analysis is one of the bio-monitoring tools addressing aquatic contamination, according to^18^. Moreover, histological studies provide data on the histopathological changes occurring in fish tissues associated with heavy metal contamination^19^. Hence, histopathology may be used as a biomarker to examine particular vital organs and to address the environment as well^18,20^.

The seasonal variations occurring in the Nile tilapia’s physiological, biochemical, and antioxidant responses to heavy metal pollution were investigated in the current study, highlighting novel insights into the adaptive mechanisms. In addition, it comprehensively evaluated hormonal regulation, electrolyte and metabolic balance, liver and kidney function, oxidative stress and lipid peroxidation levels, along with histopathological changes in the gills, liver and kidney providing an integrated understanding of how heavy metals affect fish health across different environmental conditions.

## Materials and methods

### Study sites

**Fig. 1:**
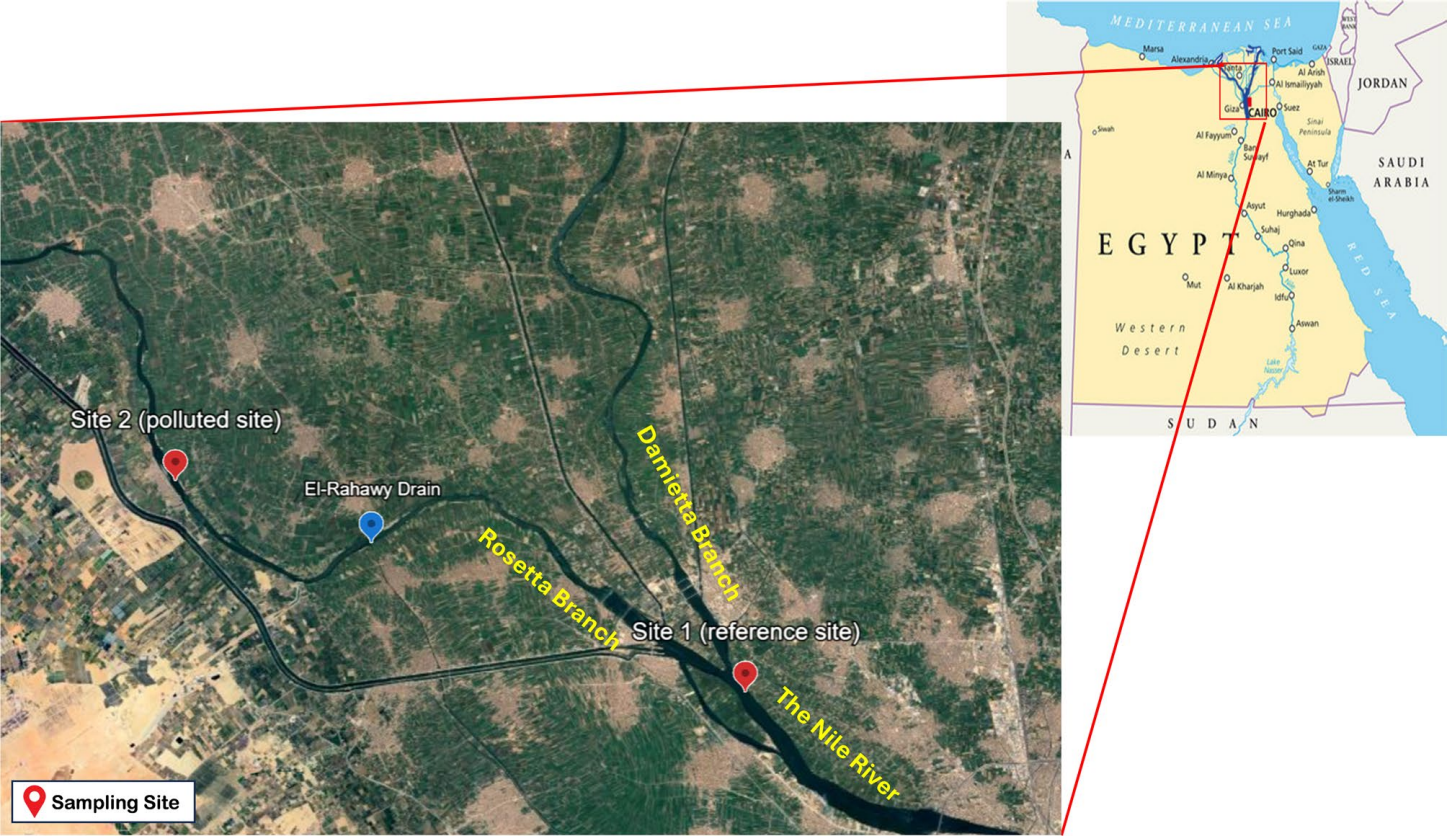
A Satellite Image of the Delta Area of the River Nile Showing Stations Under Study.

The River Nile’s Rosetta Branch is approximately 225 km long, 150–200 m wide, and has an average depth of 2.0–3.5 m. It extends from El-Qanater El-Khayria to the Rosetta Estuary. The current study targeted El-Rahawy drain, which collects agricultural, industrial, and domestic pollutants from the Giza City. More than 1.9 million m^3^ of sewage wastes are daily discharged into the Rosetta Branch of the River Nile^2^. The study area covers a distance of roughly 20 km from El-Qanater El-Khayria station to El-Qatta station, with S1: El-Qanater El-Khayria station being the reference clean site before the bifurcation, and S2: El- Qatta station (about 12.6 km from Site 1) which is approximately 7 km from the Rahawy drain’s discharge point into the Rosetta Branch. The map representing the sampling sites was generated using Scribble Maps (https://www.scribblemaps.com/), an online mapping tool, with geographic coordinates based on field data, using Mapbox Terrain base map (Fig. 1).

### Fish sampling

#### Sample collection

##### Water and fish sampling

Water and fish samples were collected twice from two selected sites during summer and winter seasons (September and December), 2023 in triplicates. The sampling was done in agreement with the ethical guidelines of the National Institute of Oceanography and Fisheries (Egypt) under the approval code: NIOF-FW3-F-23-R-003. Surface water samples were collected at a depth of 30 cm from each site. The Physico-chemical parameters of water were appraised according to the standard methods set by the **APHA**^21^. Parameters included dissolved oxygen (DO), pH, electrical conductivity (EC), and water temperature, measured using a multiparameter (Hanna HI9829, Woonsocket, RI, USA).

A total number of forty Nile tilapia specimens were sampled, with a mean total length and weight of 20.03 ± 1.62 cm and 163.19 ± 24.08 g, respectively. Fish were collected with the help of fishermen from two stations: El-Qanater El-Khayria (reference Site) (S1) and El-Qatta (polluted site, S2, affected by El-Rahawy drain). El-Qatta was selected being the first location where fish are allocated just after the discharge point of El-Rahawy drain. The blue mark denotes El-Rahawy drain, identified as the primary source of pollution across the study area^2^.

At the end of the experiment, fish anesthesia and euthanasia were conducted following the protocol described by Fernandes^22^, using pure clove oil (90–95% eugenol) obtained from Magnus Mabec and Reynard, USA. Fish were anesthetized by immersion in a clove oil bath at a concentration of 0.1mL/ L for five minutes. Anesthetic induction was confirmed by observing some characteristic symptoms, viz. the loss of equilibrium, reduced opercular movement, and decreased pectoral fin activity. After blood sample collection, euthanasia was performed by re-immersing the fish in a clove oil bath at 0.4mL/ L for five minutes to ensure a humane and rapid loss of consciousness prior to subsequent laboratory analyses.

Samples of edible tissues (muscles) were dismembered from each fish to appraise the HM contents (Zn, Cu, Cd, Fe, and Cr), according to **APHA**^21^ by the atomic absorption reader (model: GBC Savant with Graphite Furnace 5000), as reported in the first published part of the current work^10^.

**Table 1:** Mean bioaccumulation of heavy metals (µg/ g) in the muscles of nile tilapia collected from two sites of the Rosetta branch during the study, as reported in the first published part of this study^10^.

HM (µg/ g)	S1 (reference site)		S2 (polluted site)		PLs
	Summer	Winter	Summer	Winter	
Fe	47.97	84.53	109.32*	186.05*	100
Zn	23.50	32.50	36.87	50.92*	40
Cu	2.25	3.62	10.59	20.33	30
Cr	0.78	1.17*	1.26*	2.09*	1
Cd	0.42	0.47	0.74*	1.23*	0.5

Each value illustrates the mean level ± SE, SE: Standard error (*n* = 10). PLs; permissible limits according to WHO^23^ except for Cr which is in accordance with the FAO^24^. *: values exceeded the permissible limits.

### Hormonal assessments

Hormones levels were evaluated using ELISA kits manufactured by MyBioSource. The catalogue numbers of cortisol and thyroid-stimulating hormone (TSH), T3 (triiodothyronine) and T4 (thyroxine) kits were (MBS704055, MBS282744, MBS2700145, andMBS701162, respectively).

### Blood electrolytes

Both serum potassium (K+) and sodium (Na+) levels were evaluated using kits supplied by Spectrum Diagnostics (Cairo, Egypt), according to the method of^25,26^, respectively.

### Biochemical parameters

The levels of serum glucose, total proteins, and albumin were assessed by colorimetric method designated by^27,28^, respectively. The enzymatic activity of alanine aminotransferase (ALT) and aspartate aminotransferase (AST) was evaluated based on the Kinetic method of^29^. Serum levels of urea and uric acid were analysed enzymatically following^30^. Serum creatinine was quantified as stated by^31^.

### Antioxidants and lipid peroxidation

The activity of superoxide dismutase (SOD) in serum was measured using the enzyme potential to supress the reduction of nitroblue tetrazolium dye according to^32^. Catalase (CAT) serum activity was evaluated colorimetrically based on the method of^33^. Glutathione peroxidase (GPx), glutathione s-transferase (GST), glutathione reduced (GR) and reduced glutathione (GSH) activities were assessed using the method described by^34–36^, and^37^, respectively. Lipid peroxidation (LPO) was estimated using the thiobarbituric acid (TBA) reaction with malondialdehyde (MDA), as cited by^38^.

### Histological studies

Five Nile tilapia samples were randomly chosen from each site; small pieces of the kidney, liver, and gills were dissected and kept in aqueous Bouin’s fluid for 24 h. Ethyl alcohol with progressive grade was used to dehydrate samples following fixation and washing. Afterwards, tissues were cleaned and put in a clear molten parablast. Haematoxylin and eosin were used to dye sections of 5–6 μm thickness^39^. The investigation was performed using a light microscope and a digital camera at the Faculty of Agriculture, Research Park, Cairo University.

### Statistical analysis

The site and season effect on the HM content in the tissues of the Nile tilapia were determined by employing Jeffreys’s Amazing Statistics Program (JASP 0.16.4) to apply two-way analysis of variance (ANOVA) on the data of the HM levels in the tissues, considering two factors: sampling site and season. Followed by post-hoc Tukey’s test to illustrate the differences between the study groups.

## Results

The findings of this study reported significant alteration correlated pollutants with both site and season, highlighting the profound dual impact of pollutants and seasonal changes on the physiological condition of fish. In Table 1, the average HM levels in the Nile tilapia recorded a site- and season-related effect, based on the HM levels and bioaccumulation in water and fish muscles, as cited in our previous, published part of the current study^10^.

The physico-chemical parameters of water samples in Table 2 illustrate a significant variation between the reference site and the polluted site across seasons. While both pH and temperature reveal non-statistical difference (*P* > 0.05) among data, the EC and DO values recorded a remarkable difference (*P* < 0.05). EC was reported with a substantial increase at the polluted site compared to the reference site during the study, exceeding the aquatic life guidelines^40^. On the other hand, although the DO shows a significant reduction (*P* < 0.05) at the polluted site, relative to the reference site, it remains within the guidelines^40^.

**Table 2:** Seasonal variations in physical and chemical water quality parameters at two sites in the Rosetta branch of the nile River.

Parameter	Reference site (S1)		Polluted site (S2)		Aquatic life guidelines
	Summer	Winter	Summer	Winter	
pH	7.85 ± 0.10^a^	8.06 ± 0.12^b^	7.53 ± 0.12^a^	7.26 ± 0.11^b^	6.0 to 8.5
Temperature (°C)	29.86 ± 0.11^a^	18.31 ± 0.24^a^	30.10 ± 0.09^a^	18.96 ± 0.27^a^	10 °C and 30 °C
EC (μS/cm)	395 ± 18.87^a^	412 ± 7.49^b^	739 ± 21.57^c^	820 ± 13.92^d^	< 500 mg/L
DO (mg/L)	8.51 ± 0.17^b^	9.22 ± 0.21^a^	7.23 ± 0.12^c^	7.45 ± 0.09^c^	>5 mg/L

Each value represents the mean ± SE, SE: Standard error (*n*=6). Values in the same row with varied superscripts are significantly different (*P*< 0.05) by Tukey’s test. Aquatic guidelines according to (Tarzwell, 1956).

### Hormonal profile

Cortisol levels exhibited a notable enhancement (*P* < 0.05) in fish from the polluted site compared to those from the reference site. Conversely, thyroid hormones (T3, T4, and TSH) displayed a substantial decline (*P* < 0.05) in fish from the polluted site, as illustrated in Table 3 These hormonal alterations were more prominent during the cold season compared to summer (Table 3).

**Table 3:** Seasonal variations in serum hormone levels of *O. niloticus* from reference and polluted sites of the Rosetta Branch.

Parameter	Reference site (S1)		Polluted site (S2)	
	Summer	Winter	Summer	Winter
Cortisol (µg/mL)	6.82 ± 0.75^c^	8.48 ± 0.67^b^	8.31 ± 0.72^b^	11.87 ± 1.1^a^
T3 (ng/mL)	37.52 ± 18.4^a^	32.66 ± 15.4^b^	30.82 ± 13.4^b^	25.21 ± 14.7^c^
T4 (ng/mL)	156.55 ± 15.3^a^	133.89 ± 14.2^b^	129.56 ± 12.5^b^	112.67 ± 8.4^c^
TSH (µIU/L)	4.81 ± 0.9^a^	4.13 ± 0.52^b^	3.37 ± 0.57^c^	2.14 ± 0.62^d^

Each value represents the mean ± SE, SE: Standard error (*n*=10). Values in the same row with varied superscripts are significantly different (*P*< 0.05) by Tukey’s test.

### Electrolyte balance

Serum sodium (Na^+^) and potassium (K^+^) levels were significantly higher (*P* < 0.05) in fish from the polluted site, while the most distinct increase occurred during winter (Table 4).

**Table 4:** Seasonal variations in serum electrolytes of *O. niloticus* from reference and polluted sites of the Rosetta Branch.

Parameter	Reference site (S1)		Polluted site (S2)	
	Summer	Winter	Summer	Winter
Na+ (μg/mL)	125.5 ± 3.6^c^	138.2 ± 2.3^b^	140.3 ± 5.2^b^	152.6 ± 3.1^a^
K+ (μg/mL)	4.21 ± 0.2^d^	5.97 ± 0.3^c^	6.77 ± 0.3^b^	8.87 ± 0.2^a^

Each value represents the mean ± SE, SE: Standard error (*n* = 10). Values in the same row with varied superscripts are significantly different (*P*< 0.05) by Tukey’s test.

### Metabolites and liver markers

Serum glucose levels in fish from the polluted site were notably elevated (*P* < 0.05) compared to those from the reference site. A similar pattern was detected in serum total protein and albumin levels, which were also increased (*P* < 0.05) in polluted fish (Table 5). Liver enzyme activity (ALT and AST) was remarkably escalated (*P* < 0.05) in fish from the polluted site, particularly in winter, compared to fish from the reference site (Table 5).

**Table 5:** Seasonal variations in serum biochemical indices of *O. niloticus* from reference and polluted sites of the Rosetta Branch.

	Reference site (S1)		Polluted site (S2)	
	Summer	Winter	Summer	Winter
Glucose (mg/dL)	71.35 ± 2.3^c^	109.21 ± 3.6^b^	94.82 ± 3.1^b^	127.93 ± 1.1^a^
Total proteins (mg/dL)	4.2 ± 0.5^c^	5.11 ± 0.4^b^	6.04 ± 0.3^a^	7.45 ± 0.2^a^
Albumin (mg/dL)	2.34 ± 0.3^c^	3.48 ± 0.2^b^	3.11 ± 0.2^b^	5.13 ± 0.4^a^
ALT (μ/L)	22.47 ± 0.7^c^	29.4 ± 0.5^b^	27.33 ± 0.3^b^	32.65 ± 0.3^a^
AST (μ/L)	6.54 ± 0.4^c^	7.68 ± 0.3^b^	7.28 ± 0.2^b^	9.16 ± 0.5^a^
Urea (mg/dL)	5.11 ± 0.2^c^	5.97 ± 0.2^b^	5.76 ± 0.2^b^	7.12 ± 0.3^a^
uric acid (mg/dL)	1.33 ± 0.09^c^	2.1 ± 0.1^b^	3.52 ± 0.07^a^	5.16 ± 0.1^a^
creatinine (mg/dL)	0.35 ± 0.03^c^	0.55 ± 0.03^b^	0.53 ± 0.02^b^	0.67 ± 0.04^a^

Each value represents the mean ± SE, SE: Standard error (*n* = 10). Values in the same row with varied superscripts are significantly different (*P*< 0.05) by Tukey’s test.

### Kidney function biomarkers

Kidney function indicators, including urea, uric acid, and creatinine, showed substantial site- and season-related elevation. Fish from the polluted site (Qatta) revealed markedly higher (*P* < 0.05) kidney function markers, especially during winter (Table 5).

### Antioxidant system activity

**Fig. 2:**
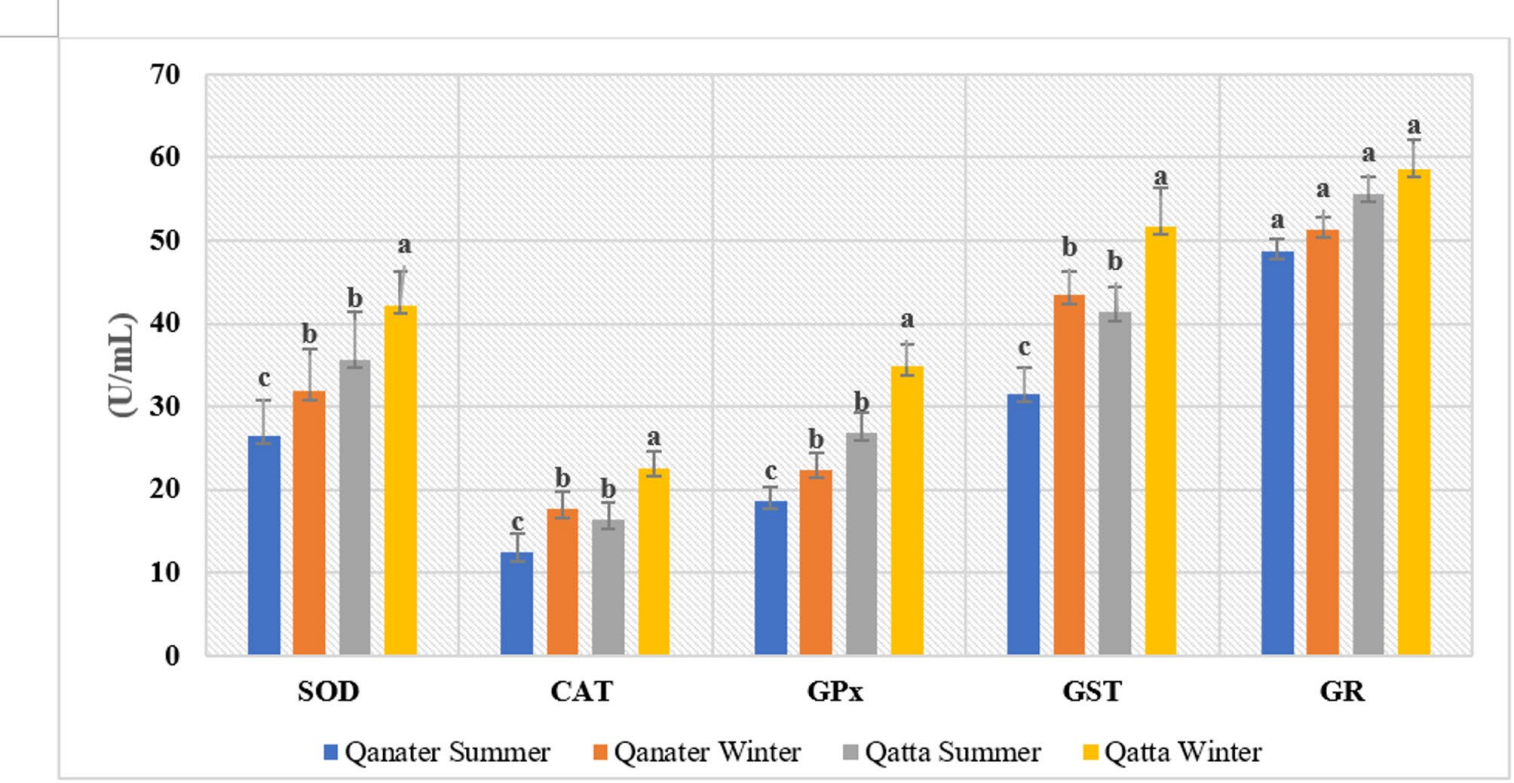
Seasonal Variations in Serum Enzymatic Antioxidant of *O. niloticus* from Reference and Polluted Sites of the Rosetta Branch.

**Fig. 3:**
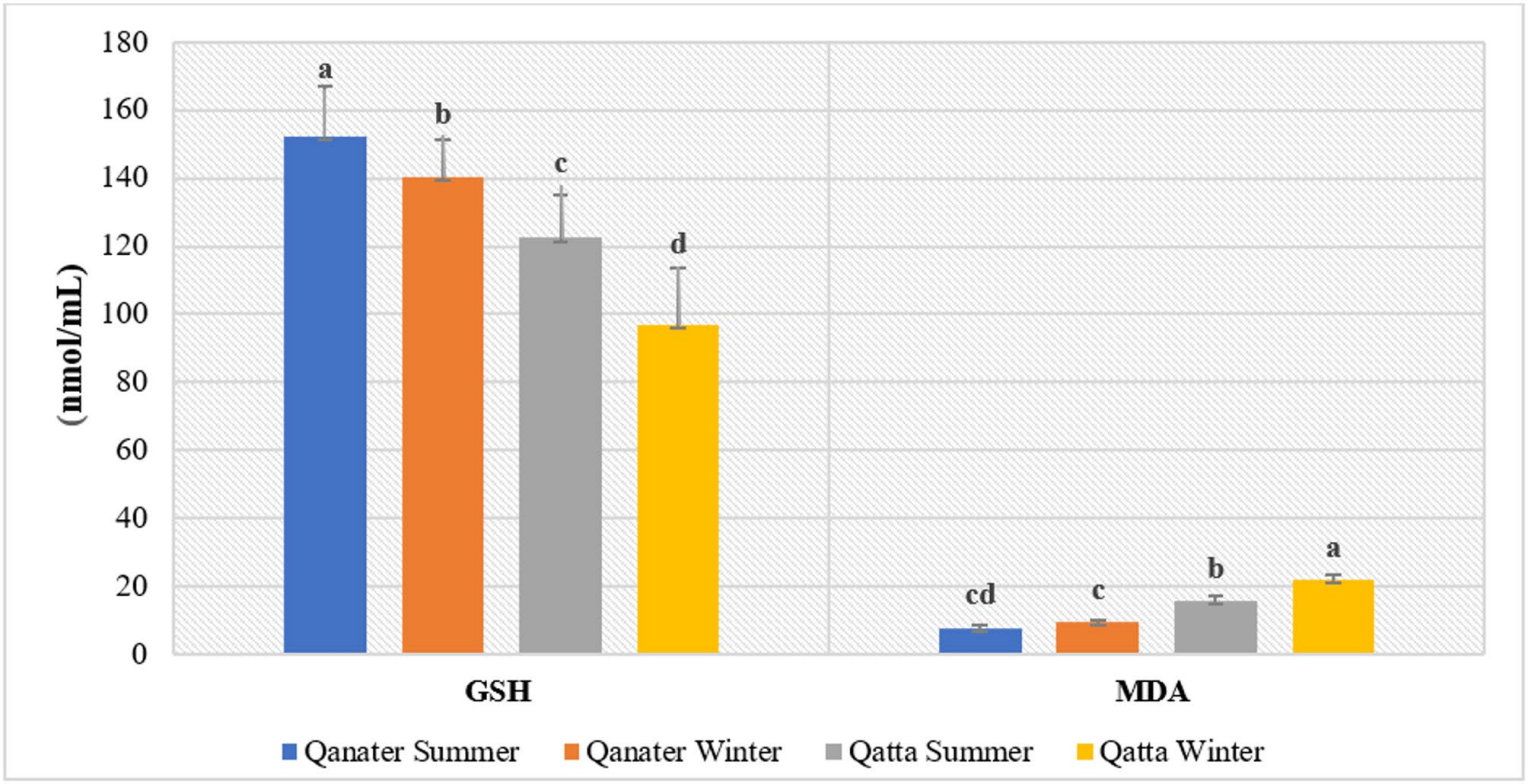
Seasonal Variations in Serum Reduced Glutathione (GSH) and Malondialdehyde (MDA) of *O. niloticus* from Reference and Polluted Sites of the Rosetta Branch.

The antioxidant defense system confirmed both site- and season-dependent variations. SOD levels were profoundly increased (*P* < 0.05) in fish from the polluted site, particularly in winter. Similarly, the levels of CAT, GPx, and GST exhibited a significant elevation (*P* < 0.05) in fish from the polluted site relative to those from the reference site, specifically in winter (Fig. 2). Alternatively, GR levels revealed a statistically insignificant elevation (*P* > 0.05) in fish from the polluted site. However, GSH levels showed a substantial decline (*P* < 0.05), particularly in winter, contradicted with fish from the reference site (Fig. 3).

**Fig. 4:**
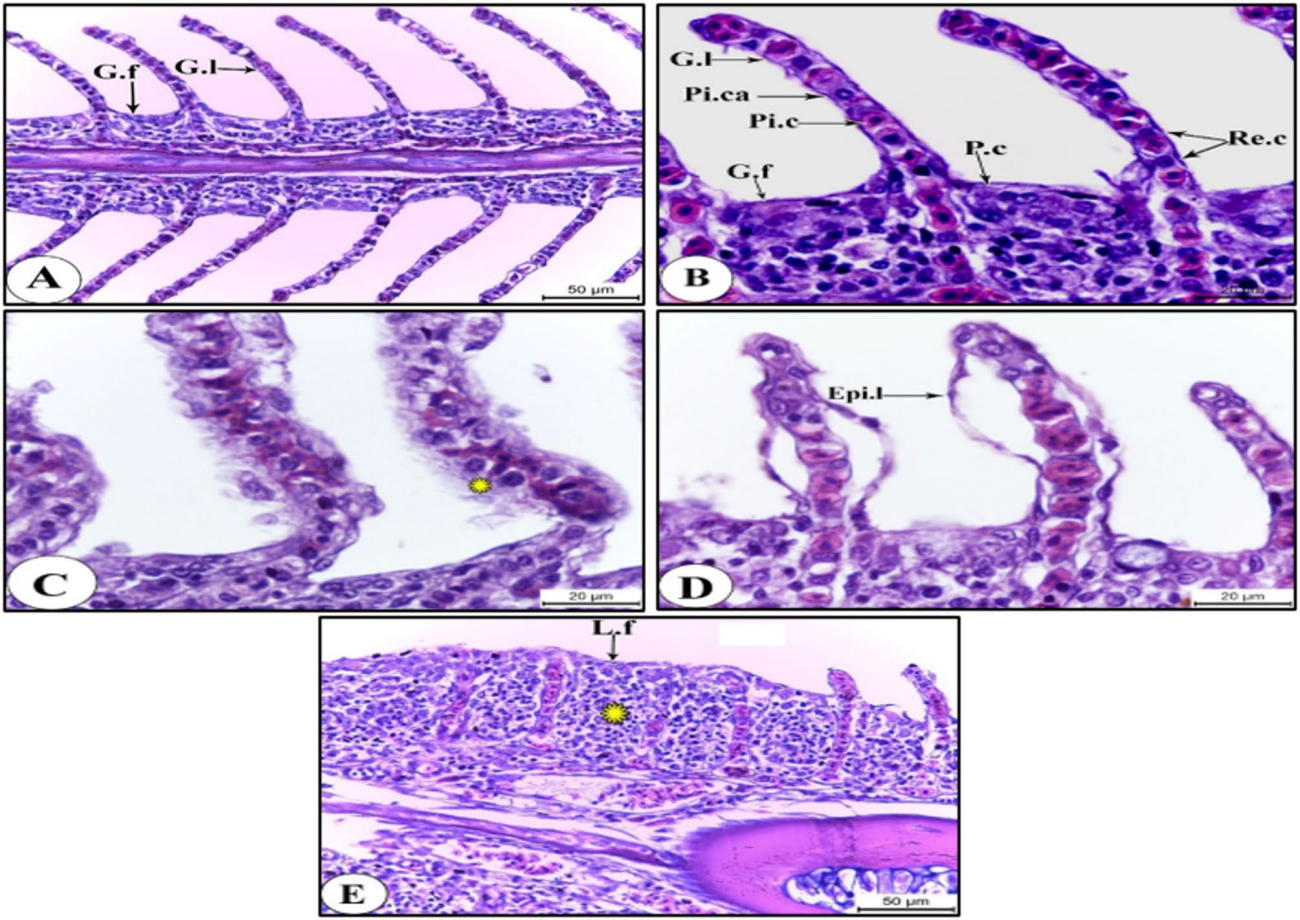
Photomicrographs of Sagittal Sections of Gills of *O. niloticus* Showing: (A&B) Normal histological architecture of the gills at S1. (C) Hyperplasia and degeneration of the respiratory cells (asterisk) at S3. (D) Epithelial lifting at S3. (E) Complete lamellar fusion with gill filament hypertrophy (asterisk) at S3. HX & E stain. Abbreviations: G.f, gill filament; G.l, gill lamella; Epi.l, epithelial lifting; L.f, lamellar fusion; P.c, pavement cell; Pi.ca, pillar capillary; Pi.c, pillar cell; Re.c, respiratory cell.

### Lipid peroxidation

The lipid peroxidation byproduct malondialdehyde (MDA) was detected with a significant elevation (*P* < 0.05) in fish from the polluted site compared to reference fish, with the most distinct increase recorded in winter (Fig. 3).

### Histological studies on selected organs of the nile tilapia

It is worth mentioning that prominent alterations in the histological architecture of the studied organs were detected during winter based on our previous findings^10^ of the poor water quality and high aggregation of heavy metals (Fe, Zn, Cu, Cr and Cd) during this season. The most prominent features were as follows;

### The gills

At the clean reference site (El-Qanater), the gills of the Nile tilapia displayed normal architecture. They are made up of a lot of gill filaments on which a bunch of gill lamellae are protruded (Fig. 4A). Pavement cells, which are stratified epithelial cells that appear as polygonal cells with a central nucleus and homogenous acidophilic cytoplasm, line the gill filaments (Fig. 4B). Simple squamous respiratory cells make up the lamellar epithelium that lines the gill lamellae. A median vascular core, or pillar capillary, divided by pillar cells, makes up the core of each gill lamella. The pillar cells are cuboidal in shape with centrally located nuclei (Fig. 4B).

On the other hand, at polluted site, the gills of the Nile tilapia showed numerous histopathological changes, such as hyperplasia and degeneration of the respiratory cells (Fig. 4C) and epithelial lifting (Fig. 4D), complete lamellar fusion (Fig. 4E) and hypertrophy of the gill filaments (Fig. 4E). Such histopathological alterations may be ascribed to the deterioration of the water quality at S3 as well as the accumulation of Cd, Zn, Fe and Cr in the gills’ tissues of the Nile tilapia at polluted station, as mentioned in our previous study^10^, recording values which surpassed the permissible levels set by the **WHO**^23^.

### The liver

**Fig. 5:**
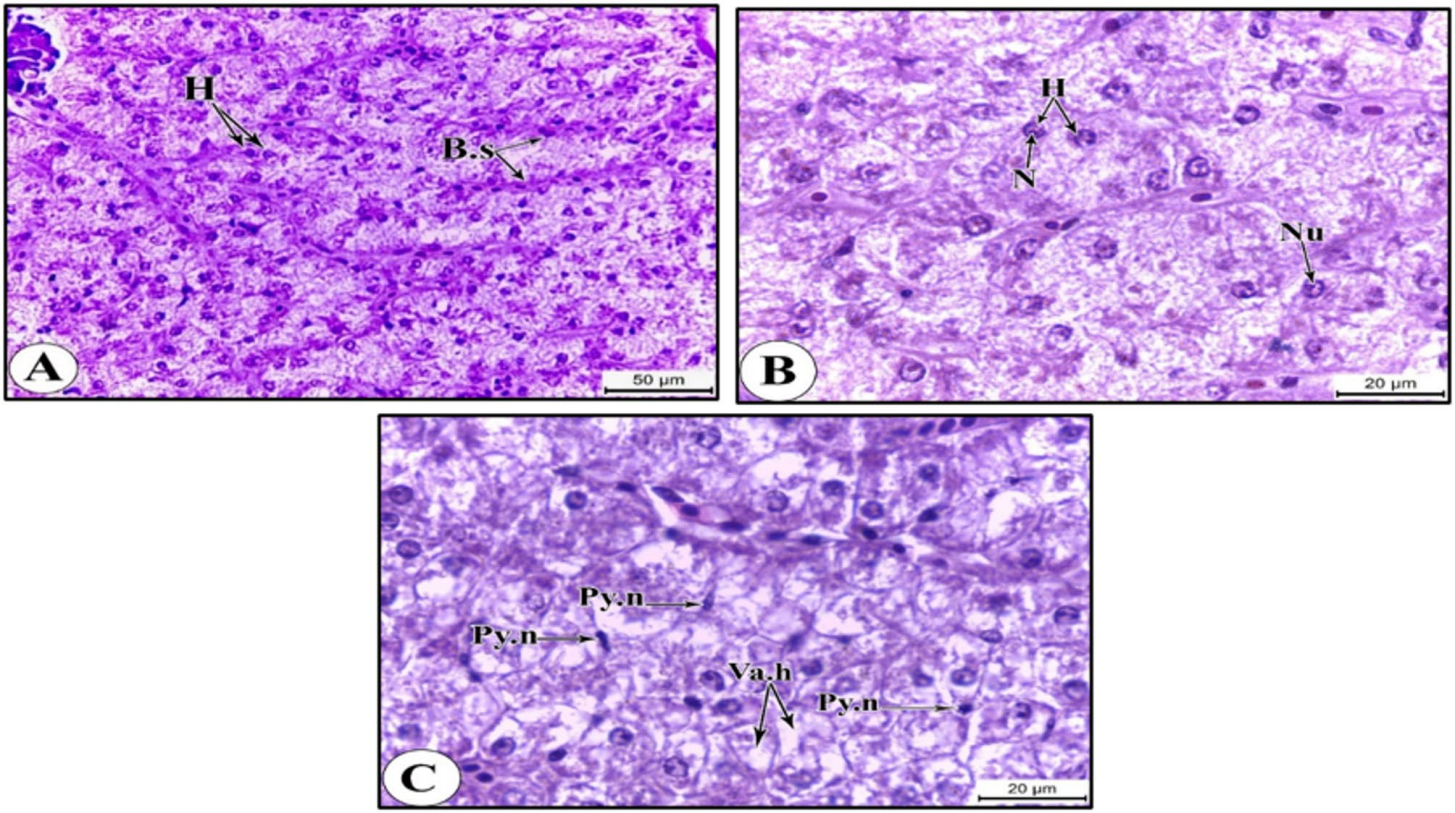
Photomicrographs of sections of Liver of the Nile tilapia showing: (A) overall normal structure of hepatocytes and blood sinusoids at S1. (B) Normal hepatocytes with nuclei and nucleoli at S1. (C) Vacuolated hepatocytes and pyknotic nuclei at S3. HX & E stain. Abbreviations: B.s, blood sinusoids; H, hepatocyte; Py.n, pyknotic nucleus; N, nucleus; Nu, nucleolus; Va.h, vacuolated hepatocyte.

The liver of the Nile tilapia specimens collected from the reference (unpolluted) site displayed a typical histological architecture. It consists of a compact mass of hepatocytes interrupted by blood sinusoids (Fig. 5A). The hepatocytes are polygonal in shape and have an acidophilic cytoplasm with a granular appearance, a large, rounded, and central basophilic nucleus, and a solitary, dark eccentric nucleolus (Fig. 5B).

On the contrary, the liver sections of the samples collected from polluted site showed histopathological alterations, such as degeneration of hepatocytes, evidenced by the vacuolation of the hepatocytes and pyknotic nuclei (Fig. 5C). Such histological malformation may be assigned to the compromised water quality of the polluted site aligned with the accumulation of Fe, Zn, Cu, Cd and Cr, previously addressed^10^, in the liver tissues of the Nile tilapia, recording values that outperformed the permissible doses recommended by the **WHO**^23^.

### The kidney

**Fig. 6:**
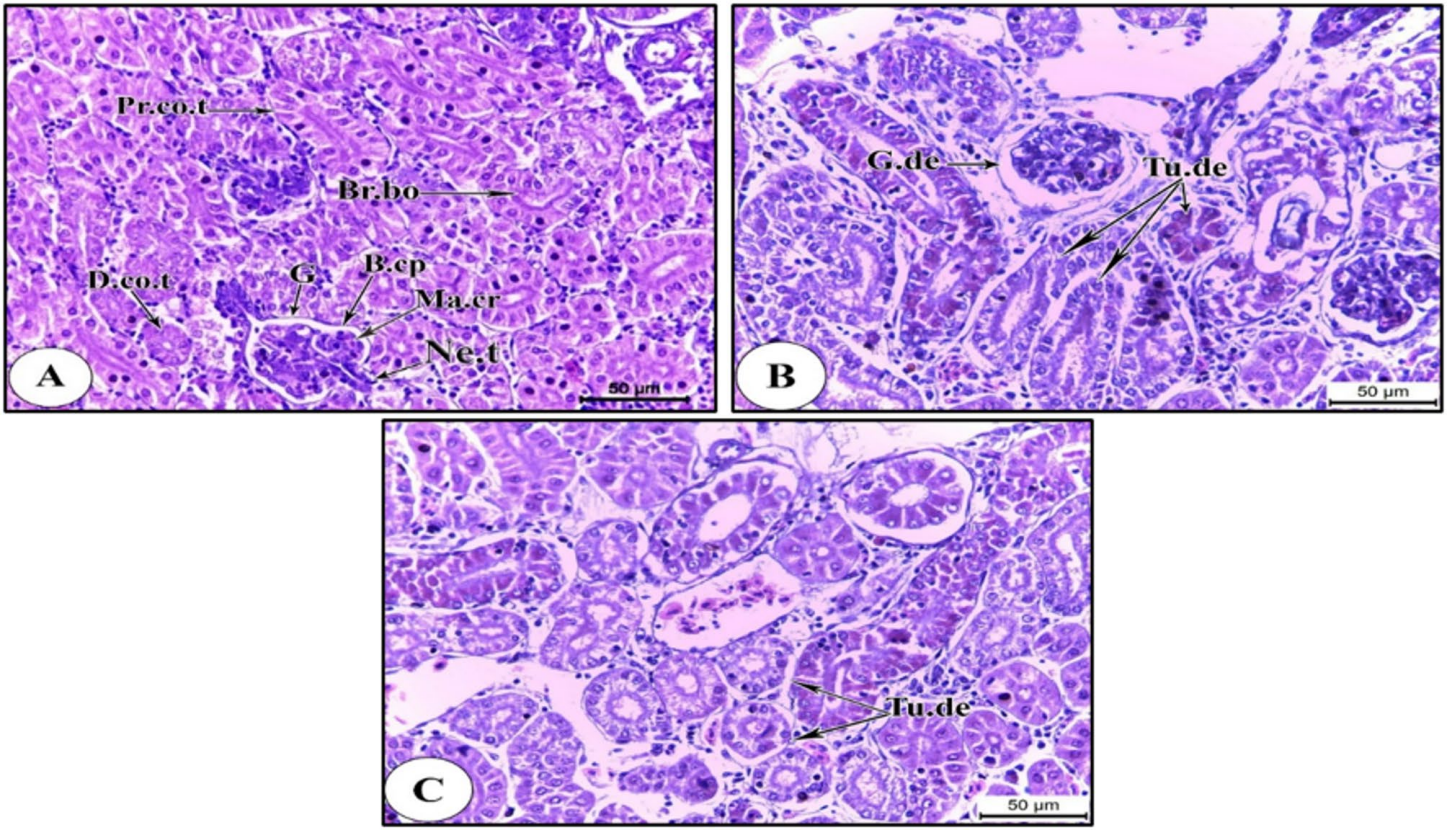
Photomicrographs of transverse sections of kidneys of *O. niloticus* showing: (A) Normal kidney structure at S1. (B) Tubular and glomerular degeneration at S3. (C) Tubular degeneration at S3. HX & E stain. Abbreviations: B.cp, Bowman’s capsule; Br.bo, Brush border; D.co.t, distal convoluted tubule; G, glomerulus; G.de, glomerular degeneration; Ma.cr, Malpigian corpuscle; Ne.t, neck tubule; Pc.t, proximal convoluted tubule; Tu.de, tubular degeneration.

The Nile tilapia kidneys that were collected from the reference site exhibited normal histological architecture. Nephrons, the functional units of the kidney, are present in large numbers in the kidney^41^. The Malpighian corpuscle, short neck tubule, proximal convoluted tubule, and distal convoluted tubule make up these nephrons. The Bowman’s capsule encloses a highly vascularized glomerulus that makes up the Malpighian corpuscle. A cuboidal epithelium with uniformly eosinophilic cytoplasm and large, spherical nuclei is positioned in the centre which lines the neck tubule. Pyramidal epithelial cells with brush borders line the proximal convoluted tubules, while short pyramidal epithelial cells without brush borders line the distal convoluted tubules (Fig. 6A).

On the other hand, the kidneys of the Nile tilapia collected from polluted site showed histopathological abnormalities, including Malpighian corpuscles degeneration (Fig. 6B) and tubular degeneration (Fig. 6B & C), due to the strong interaction between kidney and water toxins. This histopathological alteration may be attributed to the poor quality of water at the contaminated site and the effect of heavy metals.

## Discussion

Blood indicators are widely recognized as valuable tools for assessing an organism’s health and understanding the toxicological impacts of pollutants. Our findings are consistent with the HM levels and accumulation reported in the first published-part of the present work^10^. These findings underscore the significant influence of HM pollution on several blood biochemical parameters in fish, highlighting the necessity of continuous monitoring and effective pollution management strategies.

Water parameters represent a crucial key for assessing the status of aquatic life, especially fish. pH is a major water parameter, affecting both the cellular biological and chemical reactions in fish and regulates metal ions’ solubility, affecting the natural aquatic environment^42^. Temperature is another critical water parameter; it controls the physiology, immunity, and fish distribution and behavior^43^.

EC expresses the capacity of aqueous solution to transmit the electrical current, solution of most inorganic compounds, and more abundant ions have higher conductivity. The upsurge in EC at the polluted site is mostly related to the domestic, sewage, and agricultural wastes discharged from El-Rahawy drain^2^. Remarkably, DO is critical for the vigorous aquatic network since low levels causes hypoxia, mortality and an enormous shift in fish populations^44^. The low DO levels detected at the polluted site confirm the prolonged influence of El-Rahawy drain, which releases inorganic and organic contaminants into the Rossetta Branch water. These pollutants consume the waters’ DO during the nitrogenous compounds’ oxidation, especially during high temperature seasons^2^.

The seasonal closure of the High Dam in Aswan mainly causes the reported deteriorated water quality and higher HM levels in the Rosetta Branch of the Nile River during winter. This, in turn, lowers both the water levels and flow in the mainstream and branches of the Nile River. This reduction limits the dilution capacity of the river, concentrating pollutants discharged from El-Rahawy drain, deteriorating the water quality, and accumulating toxins in fish. This seasonal, hydrological effect compounds the higher toxic discharge loads reported in winter, all contributing to elevated toxicity levels observed in fish during that season^2^.

In the present study, HM exposure led to a significant increase in blood glucose, signifying metabolic change^45^. In fish, variation in blood glucose levels is commonly adopted as an indicator of stress response. The observed hyperglycaemia is likely linked to HM pollution, as HMs are known to alter carbohydrate metabolism in fish, promoting glucose synthesis from extrahepatic tissues such as proteins and their constituent amino acids^46^. Additionally, fish exposed to stressful environments tend to expend more energy to cope with stress, consequently exhibiting higher serum glucose levels^47^.

Serum proteins play a key role in the transport of HMs^48^. Consequently, the effect of HMs on total serum protein levels is regularly used to understand fish responses to environmental stressors^49^. The primary blood protein, albumin, is fundamental for the transportation of both exogenous and indigenous substances, in addition to controlling blood osmotic pressure. Since most serum proteins are synthesized in the hepatocytes, elevated total serum proteins and albumin in this study proposes liver malfunction induced by HM pollution, in accordance with^2,50^.

The observed hyperlipidaemia in the Nile tilapia from the polluted site may be associated with liver and lipid metabolism dysfunction. Lipids are markedly crucial constituents of cell membranes, which sustain cellular fluidity; therefore, membrane injury caused by HMs could promote hyperlipidaemia^51^. In this context^52^, stated that stress induced by HMs increased lipid mobilization to cope the rising energy demands in fish, which aligns with the hyperglycaemic response detected in this study. ALT and AST activities in serum are utilized as stress-sensitive indicators in fish, and their recorded substantial escalation advocates stress-induced liver damage. A comparable elevation has been stated in the plasma and serum of fish subjected to HMs, assigned to hepatocyte injury, which subsequently caused cytosolic enzymes’ leakage into the bloodstream^2^.

The kidney is known to play a significant part in the detoxification and discharge of toxicants. Urea, uric acid, and creatinine are non-protein nitrogenous compounds, while creatinine is a waste product mainly from muscles breakdown, urea is the major metabolite from dietary protein and tissue protein turnover. Uric acid in fish is formed from exogenous and endogenous purines and is then converted in the liver to urea and excreted by the gills^53^. It is worth noting that, urea, uric acid, and creatinine are useful in the diagnosis of renal dysfunction, muscle damage, and nitrogen metabolism impairment^54^.

The kidney plays a substantial role in the detoxification and elimination of poisonous substance from the body. Creatinine is a byproduct of creatine break down; a constituent that aids muscles generate energy. It is frequently adopted to evaluate kidney function. When high levels of serum creatinine are detected, kidney impairment can be assigned. Urea is eminently the main metabolite from dietary protein breakdown. In fish, uric acid is synthesized from both endogenous and exogenous purines in the liver and excreted through the gills^55^. These indices are generally used in the diagnosis of renal degeneration and muscle injury in addition to nitrogen metabolism dysfunction^56^.

The current azotaemia triggered by HMs is characterized by low blood flow and filtration rate, and is identified by high levels of serum urea, uric acid, and creatinine^57^. Both Na^+^ and K^+^ are the major electrolytes in fish; these electrolytes responsible for maintaining the acid-basic equilibrium^58^. Additionally, the homeostatic mechanisms of fish typically respond to changes in the surrounding ecosystem, hence electrolytes are employed as indicators of contamination^47^. Generally, stress and gills damage disrupt osmoregulation in fish by impacting the gills’ ability to regulate ion permeability^59^. Elevated Na^+^ and K^+^ levels indicate kidney and gill impairment, disrupting the osmoregulatory function in fish^60^. Our findings support this hypothesis, as the blood electrolyte levels observed in this study correlate with HM concentrations and the pathophysiological changes in fish from sampling sites.

Under stressful environments, fish recorded higher levels of catecholamines, such as adrenaline and noradrenaline, which enhance the levels of cortisol and corticosteroids^61^. This hormonal surge activates the negative feedback response, improving serum glucose levels, as observed in the current study. However, the release of cortisol is the main physiological response to stress, while glucose production is the secondary response^62^. In the present study, fish exposed to HMs recorded higher serum cortisol levels; similar to our findings^63,64^, observed higher cortisol and glucose levels in fish exposed to stressful conditions. In this respect^61^, reported an upsurge in cortisol levels due to Pb exposure.

Cortisol levels in fish correlate positively with HM concentrations in the neighboring environment. Giving the studies carried out by^65,66^, the influence of HM exposure has been found to inhibit the endocrine function by altering the production and metabolism of cortisol, negatively affecting the health of fish. Moreover, for cortisol^67^, the substance is eliminated in the renal pelvis and subsequently introduced into the bloodstream. Generally, fish excrete cortisol through the gills, kidneys and liver, with branchial excretion being the primary route. Factors such as water quality, HMs, and chronic stress can alter excretion rates^68^. However, the histological lesions observed in the gills and liver may impair cortisol excretion, leading to its accumulation in the bloodstream and intensifying stress-related effects.

Exposure to toxic HMs has been revealed to upsurge the production of reactive oxygen species (ROS), including superoxide anions, hydroxyl radicals, and hydrogen peroxide. This leads to a state referred to as “oxidative stress,” which can cause substantial harm to cellular structures and functions^69^.

Recently, the mechanisms and modes of action underlying HM cellular toxicity have developed as a significant area of research. Redox-active HMs, such as Fe, and Cu, are known to involve in redox cycling responses. In contrast, redox-inactive HMs, including lead (Pb) and Cd, primarily deplete key cellular antioxidants, especially thiol-containing antioxidants, such as glutathione and related enzymes. Furthermore, exposure to HMs can alter the regulatory systems, energy metabolism, and imbalance electrolyte levels in fish^70^, as deduced in the current study. Thus, the antioxidant defense system has gained significant attention as a potential early indicator of HM-induced stress, functioning as a pre-detector to identify cellular stress before severe damage occurs in fish. Antioxidant defences typically consist of both enzymatic and non-enzymatic components, including SOD, CAT, GR, GPx, and GST. However, under extreme stress conditions, the capacity of these antioxidant enzymes can be overwhelmed, leading to oxidative stress and potential cellular damage^69^.

The findings of this study confirm that the activities of antioxidant enzymes (SOD, CAT, GR, GPx, and GST) were significantly altered in the Nile tilapia off the polluted site (El-Qatta) compared to those from the reference site (El-Qanater). These changes indicate that the fish experienced physiological stress.

The antioxidant defense system has a substantial role in the response of fish to diverse stressors. SOD is a vital antioxidant enzyme in scavenging excess ROS; it changes superoxide radicals (O_2_^·^) into hydrogen peroxide (H_2_O_2_). The removal of both endobiotic and xenobiotic metabolites is predicated heavily on GST activity. Enhanced GST activity is a defensive strategy versus oxidative stress and further adverse effects of HMs. Even a meager upsurge in ROS levels in fish exposed to HMs may cause a significant damage^17^.

SOD stands as the most powerful cellular antioxidant and serves as the initial enzyme in the elimination process of ROS. It converts superoxide anion (O_2_^·^) into molecular oxygen (O₂) and hydrogen peroxide (H₂O₂). The resulting H₂O₂ is then broken down into H_2_O_2_ and O_2_ by the action of CAT, effectively continuing the detoxification process initiated by SOD. GPx, another crucial endogenous antioxidant enzyme, further converts H₂O₂ into H_2_O and changes lipid peroxides into alcohols. Among the non-enzymatic antioxidants, glutathione-related enzymes including GR and GST are significant in the detoxification process, converting countless electrophilic compounds into more water-soluble constituents, serving as secondary antioxidants and detoxification agents^69^.

Both SOD and CAT activities revealed a seasonal and site-specific variations in fish sampled. The notable increase in SOD activity in fish from polluted site (El-Qatta), especially during winter, points to a rise in O_2_^·^ caused by exposure to HMs^16^. On the other hand, CAT activity in fish from El-Qatta displayed a clear seasonal pattern; higher CAT activity indicates a response to higher oxidative stress from HMs and increased metabolic activity^5,71^, which was confirmed by the current noticeable increase in glucose and cortisol levels in fish from the contaminated site, especially in winter. However, the decline in CAT activity during winter suggests a failure in redox balance, indicating overproduction of ROS^72^.

The observed decline in GSH levels may be linked to the increased activity of GST, which utilizes GSH for the elimination of xenobiotics^73^. reported higher GST activity and expression in fish from heavily polluted areas, emphasizing its role in protecting tissues from oxidative damage. Notably, GST plays a crucial role in detoxification by facilitating the conjugation of GSH to xenobiotics, making it a key component of the oxidative stress response^74^^75^. stated that GST levels in fish were significantly increased, correlated with a decline in GSH levels, aligning with the current data. GSH levels typically decrease following exposure to toxicants due to oxidative stress but may later increase as a compensatory response^76^. In contrast, GR activity remained largely unchanged. This insensitivity of GR was also observed in earlier studies on fish^55,77^.

HMs, especially Cd, has been reported to disrupt the antioxidant defense system, draining reduced GSH and altering the activity of antioxidant enzymes^78,79^. observed elevated GSH levels and GR activity in fish exposed to HMs, highlighting the importance of GSH metabolism in mitigating HM-induced toxicity. These findings corroborate the current data, which show increased GST activity and decreased GSH levels.

Moreover, higher GPx likely contributed to advanced GSH utilization and subsequent declines in GSH levels^73^^80^. proposed that elevated GR activity enhances GSH recycling and increases the GSH/GSSG ratio^81^. demonstrated that HMs such as Pb binds to GSH, promoting ROS production. ROS detoxification involves the oxidation of GSH to GSSG by GPx, and subsequently GR reduces it back to GSH. However, if the production of the oxidized form (GSSG) surpasses GSH, it leads to the reduction of the GSH pool^82^.

Moreover, chronic exposure to HMs for the Nile tilapia resulted in elevating GPx activity, as observed in the study of^83^, coinciding with the present findings. Similarly^75^, reported a gradual reduction in GSH levels in goldfish, attributing it to sharp consumption and degradation of GSH, as part of the fish’s adaptive response. These observations agree with the data recorded at El-Qatta site under study.

Additionally, the upsurge in MDA levels at the polluted site, especially during winter, specifies noteworthy lipid peroxidation and cellular damage^5^. MDA is a marker of oxidative stress, and higher levels suggest that the antioxidant defences failed to eliminate HM-generated ROS and protect cells, particularly in winter. These findings highlight the seasonal vulnerability of fish to HM-induced oxidative stress, with winter posing a greater challenge due to the combined effects of high levels of HMs in the Nile River and low temperatures. This underscores the importance of considering seasonal fluctuations upon assessing the impact of environmental pollutants on aquatic ecosystems. Moreover, the impact of pollution was more severe during winter than in warmer seasons^84^, in this context, found that low water temperatures triggered oxidative stress and histological damage in the Nile tilapia. Similar findings were reported by^16^, who elucidated the intense stress responses in the tilapia off polluted sites along the Nile River during winter.

In fish, gills, being directly contacted to water, are the most vulnerable to water contaminants. They are regarded as the most sensitive and important organs, capable of withstanding adverse conditions from either the internal or external environment^85^. In addition to maintaining the ideal osmotic pressure and acid-base balance of physiological fluids, they are in charge of respiration^86^.

Notably, the gills are regarded as the main target for the HM toxicity^87^. The gills were detected with high concentrations of heavy metals due to the continuous, direct contact with contaminated water. Moreover, the gills are the main sites for the passage of the surrounding elements, with their high surface area and thinnest epithelium, compared to all other organs, facilitating the pathway of the HMs^87,88^. The highest metal concentrations have been noticed in the gills due to their close contact with water. The high concentrations of HMs in the gills are indicators of water contamination^89,90^. added that, during gaseous exchange, the gills enable contaminants to penetrate the body.

The present results coincide with those of^91–93^ who found that the exposure of the gills of *Salamo garidneri*, *Senegales sole*, *Sole senegolensis*, and *Auchenoglanis occidentalis*, respectively, to five heavy metals, such as Cu, Cr, Zn, Cd and Fe caused complete lamellar fusion, hyperplasia of the lamellar epithelium and epithelial lifting. The aforementioned authors explained these pathological changes as non-specific response of the gills to the toxic heavy metals and added that the goal of these reactions is to reduce the effect of toxins by increasing the diffusion distance between the water and blood.

 Related^7,87^, and^94^ the gills histopathology in the Nile tilapia to their exposure to high levels of Cu and Zn, respectively. Similar results were reported by^95^ and^96^ who found that exposure to Cd caused similar histopathological alterations in *Siganus rivulatus* and *Luciobarbus xanthopterus*, respectively. Furthermore^97^, postulated that the gills of *Solea agypti1aca* had the same histopathological alterations due to the water contamination with Cd, Zn, Cu, Cr and Fe.

Argued^98^ that exposure of the goldfish, *Carassius auratus* to CuO nanoparticles caused histopathological changes in the fish gills, including hyperplasia, lamellar fusion, and gill cell edema. Moreover, the wolf fish, *Hoplias malabaricus* experienced histological alterations, spotted in the gills, related to the presence of elevated concentrations of Fe in water. These changes included hyperplasia, secondary lamellar fusion, folding in the secondary lamella tip, and an increase in mucous cells^99^. The previously mentioned authors attributed hyperplasia and the increase in mucus cell numbers to defense mechanisms against external contaminants.

 Elucidated^100,101^ that the lamellar fusion found in the gills of both the Nile tilapia and *Clarias gariepinus*, respectively, is a result of hyperplasia of lamellar cells due to the reduction in gaseous exchange.

The hyperplasia of the respiratory cells and epithelial lifting can be considered as defense responses of the fish, as these alterations lengthen the distance that waterborne irritants, such as ammonia and microorganisms^102^, must travel to reach the blood stream^103^. Hyperplasia of the respiratory cells and epithelial lifting were observed in the gills of *Mastacembellus armatus*^104^ and *Channa punctatus*^51^ upon exposure to heavy metals.

 Revealed^88^ comparable results of lamellar fusion, hypertrophy, hyperplasia in the gills of fish. The aforementioned authors attributed these histopathological alterations to ion exchange; the mechanism that takes place when osmoregulation occurs. Therefore, metals are assumed to have significant effects on lamellar fusion and filament epithelium proliferation. Moreover, the presence of the gill hyperplasia, lamellar fusion, epithelial necrosis, and edema has all been linked to heavy metal toxicity.

The histological analysis of the gills of the Nile tilapia indicated that exposure to a Cd compound like CdCl_2_ drastically changes the histopathological profile, evidenced by primary lamellae dilation, secondary lamellae splitting, detached sub lamellae, and inter lamellar cells hyperplasia^105^. The aforementioned authors explained the epithelial lifting and the hyperplasia as an adaptive mechanism through which protective barriers are established between the external and internal environments of the fish via increasing the thickness of the epithelial cell layer. Additionally^106^, reported that Cd causes histopathological changes in the gilthead seabream, *Sparus aurata* (L.) gills, viz. hyperplasia, infiltration and epithelial lifting.

Liver is the most important organ for regulating how food is metabolized, stored, and processed^85,107^. According to the previous authors, the liver can get rid of extra harmful substances since their bioaccumulation can disrupt metabolism.

A good sign of HM pollution is the liver. As a result, its great affinity for the majority of the metals is included in the current investigation. The liver, which serves as the body’s main metabolic organ, is the organ where all hazardous compounds are finally metabolized. As a result, when all organs get free from metals, the latter accumulate in the liver and stay there for a long period of time^108^.

Cu caused hepatocyte vacuolation in *Ictaburus nebulus*^109^. Moreover, Cu and Zn intoxication was observed in the liver of *Puntius conchonius* that caused hepatocytes degeneration and cellular vacuolation^110^. Furthermore^87^, suggested that Cu caused hepatocytes vacuolation and nuclei pyknosis in *O. niloticus*. Additionally^111^, demonstrated that the liver of *Labeo rohita* showed similar histopathological alterations due to the exposure to high levels of pollutants, such as Fe, Cu, Cd, Zn, and Cr. In addition^112^, found that the exposure to Cd, Cr, Cu and Zn caused hepatocytes vacuolation in several fishes, including *Barbus babus*,* Chondrostoma nasus* and *Squalius cephalus*.

Reported^93^ that high concentrations of heavy metals, such as Zn, Fe, and Cd caused lipid and/or glycogen deposition in *Auchenoglanis occidentalis*, consequently caused the hepatic vacuolation^113^. explained that hepatocytes degeneration may be attributed to gills degeneration which reduced the oxygen content. The previous author added that Cd contamination caused different levels of hepatocytes degeneration. In our point of view, such vacuolation may be attributed due to this DNA degradation.

Suggested^95^ that hepatocytes degeneration of *Siganus rivulatus* occurred as a result of the accumulation of electrolytes and water within the hepatocytes due to Cd exposure that caused cell membrane impermeability succeeded by hepatocyte degeneration, indicated by vacuolation. Similar result was reported in *Luciobarbus xanthopterus*^96^.

Histopathological damage of the liver has been attributed to the exposure of fish to high levels of heavy metals^98^. reported that exposure of *Carassius auratus* to CuO nanoparticles caused histopathological changes in the fish liver, including vacuolation, blood sinusoid bleeding and cell necrosis. It was found that Cd, on the other hand, caused hepatic necrosis, hepatic blood vessels congestion, hepatic cell vacuolation, pyknosis, and hepatopancreatic duct necrosis in the liver of *Sparus aurata* (L.)^106^. While^99^, noticed blood congestion in sinusoids, hepatocyte vacuolization and necrosis in the liver of *H. malabaricus* associated with the exposure to high concentrations of Fe in water.

Kidney is the main organ responsible for preserving body hemostasis^107,114^. To keep body homeostasis within acceptable ranges, the kidney’s primary job is to filter body fluids from blood waste products^104^. According to^41^, the kidney has a significant interaction with the contaminants in the surrounding water. In addition, they deduced that the kidney of the cichlid fish, *Tilapia zillii* was impacted by toxins exhibiting a number of histological alterations.

^93^ reported that high concentration of Zn, Fe, and Cd caused renal tubular degeneration in *Auchenoglanis occidentalis*. Our results mimic those reported by^115,116^ who found similar histopathological changes in the kidney after the exposure of Nile tilapia to Cu, Zn and Cd^41^. reported the same histopathological changes in *Tilapia zilii* exposed to Cd, Cu, Fe and Zn^117^. found that the exposure of Tilapia spp. to Cu, Zn, Cd and Fe showed identical renal histopathological alterations.

^88^ analyzed Cd, Zn and Cu levels in *Boops boops* kidneys and found the following histopathological changes; the renal parenchyma was slightly changed, with disordered tubuli and somewhat crowded glomeruli. Moreover, inflammatory cell aggregation, renal tubule cellular integrity loss, minor renal tubule shrinkage, and renal tubule degeneration were among the alterations recorded. The aforementioned authors attributed these abnormalities to the accumulation of Cd and other heavy metals in the kidneys which might cause histopathological abnormalities and affect the organ’s detoxification mechanism^108^. added that the kidney is one of the main sites where Cd builds up, with the highest cadmium concentration. Moreover^106^, noted that increased Cd levels caused histopathological changes in seabream (*Sparus aurata* L.) kidney, including: tubular epithelial cell degeneration, glomerular tuft atrophy, and severe hemorrhage in addition to renal tube necrosis.

## Conclusion

The study confirms that heavy metal pollution induces significant physiological stress on the Nile tilapia off the Nile River, with seasonal variations intensifying these effects. Polluted fish, exhibited prominent endocrine disturbance. Electrolyte imbalances, metabolic, hepatic and renal dysfunction further highlight the harmful impact of contaminants. The oxidative stress response, marked by increased antioxidant enzyme activity and lipid peroxidation, specifies an attempt to mitigate the impairment induced by prolonged exposure of heavy metals. Histological analysis of the Nile tilapia ’s gills, liver, and kidney displayed severe structural injury in polluted areas, underscoring the damaging consequences of heavy metal contamination. These findings emphasize the importance of continuous environmental monitoring and strict pollution control measures to protect aquatic biodiversity and ecosystem health. Future research should explore the long-term ecological ramifications of these physiological disturbances and investigate the mitigation strategies to reduce pollution-related stress in aquatic organisms.

### Research limitations

A limitation of this study is that histopathological examinations were performed only during the winter season, when heavy metal levels were at the highest. This approach was based on the assumption that greater pollutant loads would produce more evident tissue alterations. However, this restriction limits the ability to assess seasonal differences in histopathological responses, and future studies should include multi-seasonal tissue analyses for a more comprehensive understanding.

## Data Availability

The datasets used and/or analyzed during the current study will be available from the corresponding author upon reasonable request.
